# The Dynamic Performance of Flexural Ultrasonic Transducers

**DOI:** 10.3390/s18010270

**Published:** 2018-01-18

**Authors:** Andrew Feeney, Lei Kang, George Rowlands, Steve Dixon

**Affiliations:** Department of Physics, University of Warwick, Coventry CV4 7AL, UK; a.feeney@warwick.ac.uk (A.F.); l.kang.1@warwick.ac.uk (L.K.); g.rowlands@warwick.ac.uk (G.R.)

**Keywords:** flexural ultrasonic transducer, air-coupled ultrasound, dynamic characterisation

## Abstract

Flexural ultrasonic transducers are principally used as proximity sensors and for industrial metrology. Their operation relies on a piezoelectric ceramic to generate a flexing of a metallic membrane, which delivers the ultrasound signal. The performance of flexural ultrasonic transducers has been largely limited to excitation through a short voltage burst signal at a designated mechanical resonance frequency. However, a steady-state amplitude response is not generated instantaneously in a flexural ultrasonic transducer from a drive excitation signal, and differences in the drive characteristics between transmitting and receiving transducers can affect the measured response. This research investigates the dynamic performance of flexural ultrasonic transducers using acoustic microphone measurements and laser Doppler vibrometry, supported by a detailed mechanical analog model, in a process which has not before been applied to the flexural ultrasonic transducer. These techniques are employed to gain insights into the physics of their vibration behaviour, vital for the optimisation of industrial ultrasound systems.

## 1. Introduction

Air-coupled ultrasound has become more prominent in recent years, due to advances in ultrasonic transducer design and fabrication [1], where more complex fabrication techniques have enabled the production of devices which are better suited to coupling with an air medium in order to propagate ultrasound signals to a target. Examples of air-coupled ultrasound applications include non-destructive testing [1,2], including the characterisation of material properties [3], wireless communication [4], and the measurement of fluid flow [5]. Transducers for air-coupled applications are often classified as either bulk or micro-machined [6]. A bulk transducer is used when the device can be coupled directly to the medium in which the ultrasound signal is being propagated, based on the requirement for the device impedance to be as closely matched as practical to that of the coupling medium. Due to the significant impedance mismatch between many piezoelectric ceramics (with acoustic impedances in the region of approximately 35 MRayl) and air (the acoustic impedance of which is around 400 Rayl), impedance matching layers are generally required, which can decrease the bandwidth of the transducer [6]. Micro-machined transducers possess improved bandwidth and coupling with air [6], and two configurations which have been successfully applied for air-coupled ultrasound include the piezoelectric micro-machined ultrasound transducer (PMUT) [7,8], and the capacitive micro-machined ultrasound transducer (CMUT), which is characterised by a wide bandwidth and excellent air-coupled performance [9,10,11]. The PMUT and CMUT contain very small and thin membranes, and are effectively flexural transducers that are driven off membrane resonance.

**Figure 1:**
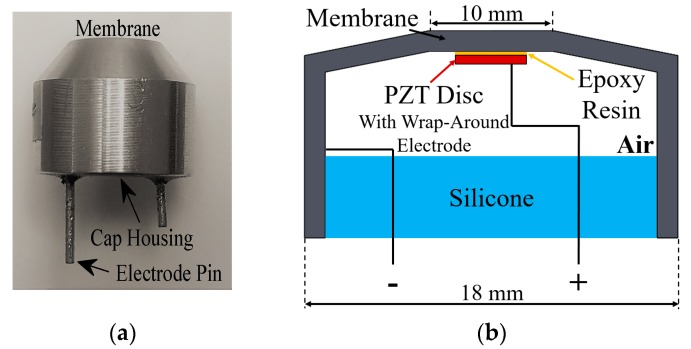
(**a**) The commercial aluminium-body FUT, showing the metal cap housing containing a piezoelectric ceramic disc bonded to the underside of the membrane, and (**b**) a side-view schematic displaying the composition of a typical FUT, where the rear of the FUT contains an air cavity with a backing layer for damping.

A relatively recent candidate for air-coupled applications at frequencies of greater than 100 kHz is the flexural ultrasonic transducer [12,13,14], which has already been utilised at frequencies below 60 kHz across a number of industry technologies, such as proximity sensing [13], and industrial measurement and metrology systems. These applications are primary targets for the flexural ultrasonic transducer (FUT), through a significantly improved understanding of the physics of their operation. The FUT, the schematic diagram for which is shown in Figure 1b and is characteristic of the type used in this study, is composed of a piezoelectric ceramic, usually in the form of a disc, bonded to a metal cap, typically composed of aluminium.

The FUT is also known as a unimorph, based on the presence of this single active layer within the device assembly. The metal cap of the FUT consists of a flat membrane layer connected to a cylindrical support, and the piezoelectric ceramic is bonded to the underside of the membrane layer with an epoxy resin. The application of a voltage to this piezoelectric ceramic causes a bending of the FUT cap-adhesive-piezoelectric composite region, generating the vibration motion which produces the ultrasound signal. When a FUT is used as a sensor, the device receives ultrasound energy which produces a bending of the membrane layer, from where it is converted to electrical energy through the piezoelectric effect. As energy is supplied to the piezoelectric ceramic, for example a lead zirconate titanate such as PZT-5H, the high frequency vibrations generate a bending of the compliant membrane layer, which can be considered as a circular plate. The resonant modes of thin circular plates can be calculated [15], where the vibration of the piezoelectric ceramic generates the plate modes with resonance frequencies which can be predicted using knowledge of the effective clamping condition of the membrane, the dimensions, and material specification of the cap.

**Figure 2:**
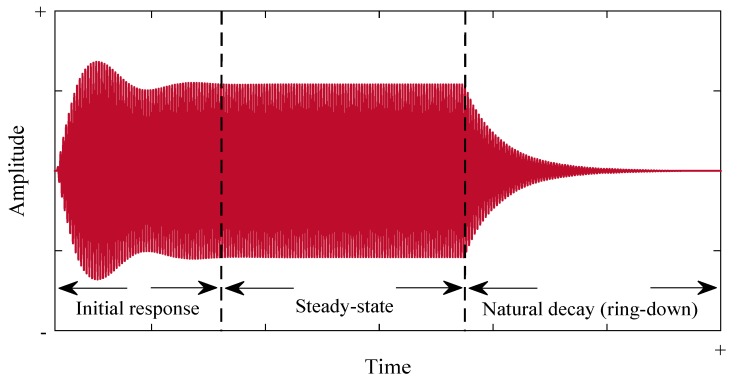
The three response regions of a FUT under forced excitation. Note that in this case the FUT is being driven at a frequency a few percent higher than its natural mechanical resonance frequency, leading to the oscillation in amplitude and larger amplitude vibration in the initial response than in the steady state.

When operating a FUT, the implementation of a continuous-wave sinusoidal excitation signal will not instantaneously produce a corresponding sinusoidal vibration motion of the metallic membrane which is in phase with this signal [14]. Part of the reason for this is obvious, since the membrane assembly possesses mass and inertia and will therefore take a period of time to reach a steady state of oscillation under a constant forced vibration. In addition to an effective mass, the FUT also has mechanical properties that will combine to produce an effective elastic stiffness or compliance and an effective damping mechanism to account for energy loss. Therefore, the FUT requires a period of time in the approach to steady-state. It has been demonstrated that the vibration response of a FUT can be considered as three distinct regions [14], which are illustrated in Figure 2. After a drive signal is applied to the FUT, the vibration amplitude response as a function of time rises from zero towards the steady-state amplitude of the second region. The vibration activity within the time range from zero to steady-state can be considered as the first region of the FUT vibration response. The physics of this initial region requires consideration because the difference between the drive frequency and the natural resonance frequency of the FUT significantly affects the measured vibration response [14]. The initial region response shown in Figure 2 is characteristic of an off-resonance response, where its vibration amplitude over-shoots the steady-state amplitude. In practical application, a FUT would be ideally operated at steady-state, which constitutes the second region, as shown using the qualitative data spectrum in Figure 2. However, a temporally long drive voltage necessary to reach a steady state of operation may not be pragmatic, or the time duration of such signals may not facilitate the required measurement. In the steady state region, standard dynamic relationships concerning forced harmonic excitation are applicable [16]. Once the drive signal is stopped, a resonant decay of the FUT vibration response occurs, also known as ring-down. This is the third region, and again, classical mathematical relationships can be used to model this transducer behaviour [16], where the magnitude of system damping determines the time required for the FUT vibration response to reach zero amplitude.

Investigations into the operation of FUTs for higher frequency applications, towards 100 kHz and above, and for high temperature and pressure environments, have only recently begun [17], and also as part of the development of phased array transducers for flow measurement [18]. Currently, FUTs tend to be operated in ambient conditions to avoid damage to component materials, such as the de-poling of the piezoelectric ceramic at temperatures approaching their Curie temperature, or collapse of the FUT membrane layer resulting from exposure to high pressure levels, for example, those exceeding 2 MPa. In practical applications in flow measurement and ultrasound generation, FUTs tend to be operated without much consideration of the physics of the vibration response approaching steady-state. The cost of this is a reduction in transducer energy efficiency or a misunderstanding of the transducer response, and so an improved understanding of the dynamic performance of FUTs is essential, particularly for those systems incorporating separate FUTs acting as transmitters or receivers. In support of the experimental observations provided in this study, a detailed mathematical analog model is reported, which accurately describes the vibration response of a FUT in the initial response region. The ability of this mathematical analog to predict the initial region vibration response for a set of dynamic parameters with accuracy is demonstrated, through correlation with experimental measurements.

## 2. Materials and Methods

The analysis of FUT vibration response is conducted by comparing three experimental techniques. The first is using an acoustic microphone (BK 4138-A-015, Brüel & Kjær Sound & Vibration Measurement A/S, ‎Nærum, Denmark), the second through laser Doppler vibrometry (LDV, Polytec OFV-5000, Waldbronn, Germany), and the third by using a second FUT as a receiver. The differences in measured vibration response between these techniques is important for the design and operation of FUT systems, particularly in relation to how FUTs are operated. The FUT transducers used here were manufactured by Multicomp, while a number of other FUT manufacturers produce transducers with almost identical designs and characteristics.

**Figure 3:**
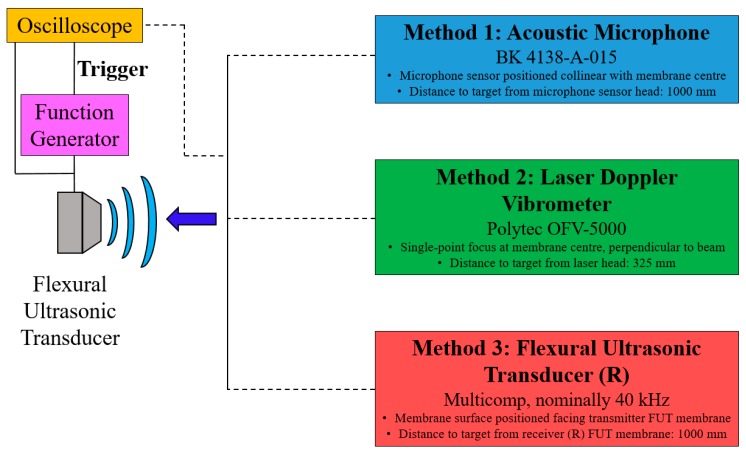
Experimental setup for the dynamic characterisation of the FUT with different sensors, and for rapid measurement of resonance frequency. Note that for a measurement of the resonance frequency of the device, the three methods shown are not required. Typical experimental parameters are also detailed.

Two aluminium-capped FUTs were used for this study. The nominal resonance frequency of the fundamental mode of the FUTs is stated to be nominally 40 kHz, but specifically reported as 40 ± 1 kHz and 39 ± 1 kHz as a transmitter and receiver, respectively. For the purposes of this investigation, despite being nominally identical, the FUT used as a receiver will be referred to as FUT_R_. The schematic of the experimental setup is shown in Figure 3, indicating how the three measurement devices can be incorporated. At a fundamental level, the experimental setup can be used to measure the resonance frequency of an ultrasonic transducer without the acoustic microphone, laser Doppler vibrometer, or FUT_R_. Using only a measurement oscilloscope and a function generator, it has been demonstrated that an expedient measurement of FUT resonance frequency can be made.

## 3. Results

### 3.1. Resonance Measurement

**Figure 4:**
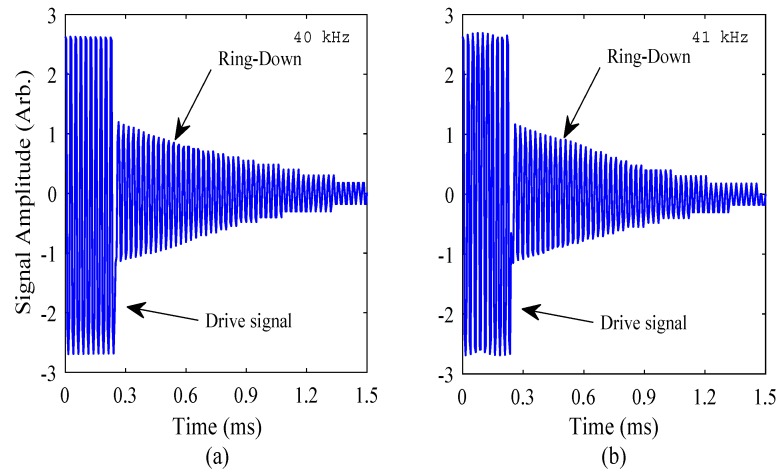
The electrical signals measured from the FUT using only a function generator and oscilloscope, for drive frequencies of (**a**) 40 kHz, and (**b**) 41 kHz.

To demonstrate the measurement of the resonance frequency of the FUT using only the function generator and oscilloscope, the Fast Fourier Transform (FFT) of the amplitude-time response was computed, from measurements recorded with drive frequencies of 40 kHz and 41 kHz, both being close to the nominal resonance frequency of the FUT. A burst sine signal with 10 cycles at 10 V_P-P_ (peak to peak voltage) was applied to the FUT, and the electrical response signal from the function generator measured using the oscilloscope. The characteristic form of these electrical signals are shown in Figure 4 for both drive frequencies, and clearly indicate the drive and ring-down regions. It should be noted that the y-axis values in Figure 4 are arbitrary in order for the ring-down region to be clearly displayed.

**Figure 5:**
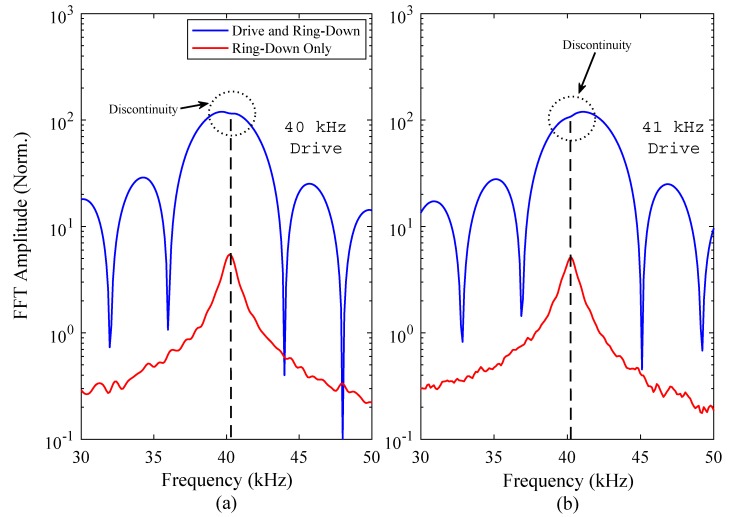
FFT results of FUT response at resonance for a 10 V_P-P_ burst sine signal of 10 cycles, showing the measurement of the resonance frequency, where the ring-down curve is superimposed, for drive frequencies of (**a**) 40 kHz, and (**b**) 41 kHz. There is a very small discontinuity, or kink, in the FFT result of each entire electrical signal of Figure 4, corresponding to the resonance frequency of the (0,0) mode of vibration of the FUT.

First, the FFT of each signal spectrum was computed, encompassing both the drive and ring-down regions measured from the oscilloscope data from the function generator. The natural resonance of the FUT for both drive frequencies was then determined, by calculating the FFT of the ring-down response region only. The FFT results are shown in Figure 5, where the response of the FUT has been split into two parts. The upper curve represents the FFT result of the total electrical response signal, comprising the drive and ring-down regions, and the lower curve shows the FFT result of the response of the resonant ring-down region only. As shown in Figure 5, a discontinuity in the magnitude of the FFT result can be identified close to the peak amplitude of each upper curve. Energy is shared between the drive signal and the natural resonance of the FUT, hence the appearance of this discontinuity in the amplitude-frequency spectrum calculated from the FFT of the measured time-dependent response.

It has been found that this discontinuity matches the centre frequency of the isolated ring-down response, which is the resonance frequency of the FUT, specifically of its closest fundamental mode of vibration—in this case the (0,0) mode. This is a key observation, as it shows that the resonance frequency of a transducer can be measured rapidly and with precision, using only basic laboratory equipment, and without the need for complex apparatus, for example an electrical impedance analyser.

Through this experiment, and the results shown in Figure 4, the resonance frequency of the FUT has been shown not to be precisely 40 kHz, but is approximately 40.3 kHz, at the drive voltage of 10 V_P-P_. This can be compared with the resonance frequency of this transducer as measured by electrical impedance analysis, which is 40.6 kHz [19]. There is a slight discrepancy between these measured values, but this can be in part explained by the effect of dynamic nonlinearity on the transducer, demonstrated in a previous study [19]. The resonance frequency of the FUT has been shown to reduce as the excitation voltage is increased, in what is termed a softening nonlinear effect. The excitation voltage used in electrical impedance analysis is typically in the order of 0.50 V_RMS_, significantly lower than the excitation voltage used to generate the results shown in Figure 4 and Figure 5. Nevertheless, the measured resonance frequency is within the stated resonance frequency range for this FUT. The identification of the resonance frequency has been made for two different drive frequencies close to resonance, where the location of the discontinuity on the amplitude-frequency spectrum gives an indication of how close the drive frequency is to resonance.

### 3.2. Dynamic Characterisation

**Figure 6:**
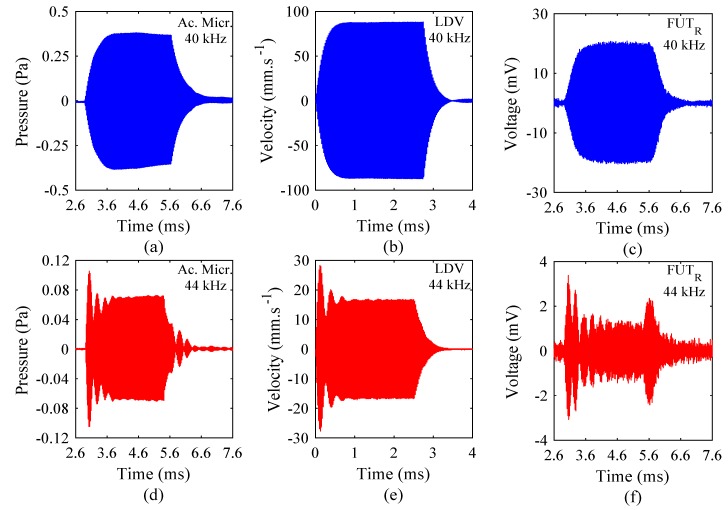
FUT response for drive frequencies of 40 kHz and 44 kHz using different measurement methods. Results for 40 kHz using (**a**) an acoustic microphone, (**b**) LDV, and (**c**) the FUT_R_ are demonstrated, followed respectively by those for 44 kHz in (**d**–**f**). The LDV results are presented from time = 0 ms to demonstrate the immediate measurement of the FUT vibration response. Note the scale changes between the graphs.

The three measurement techniques—comprising the acoustic microphone, LDV, and the FUT_R_, as shown in Figure 3—are compared in the analysis of the vibration response of the FUT, using the same set of drive conditions for each. The FUT was driven at two frequencies, one at 40 kHz, at its nominal resonance frequency, but slightly lower than the resonance frequency measured in Section 3.1, and the other at an off-resonance frequency of 44 kHz. A nominal drive excitation voltage of approximately 10 V_P-P_ was administered in each case, for a burst sine excitation of 110 cycles, with a trigger interval of 20 ms for all measurements. The acoustic microphone was connected to its dedicated amplifier system, and configured to generate 1 Pa per 1 V. A commercial amplifier (Sonemat Two Channel Echo) was incorporated in the measurement of the FUT response using the FUT_R_, in order to provide enough gain in the response signal to generate a response spectrum of sufficient resolution. The amplifier was connected directly to the FUT_R_, where the gain required to generate a signal of sufficient voltage amplitude is approximately 100. The results of these experiments are shown in Figure 6. The acoustic microphone and FUT_R_ measurement results include a time delay, which represents the time taken for the sound to travel through the air medium and be collected by the respective sensor. The reason that there is no time delay in the response measurements from the LDV experiments is that this is an optical measurement technique which provides instantaneous vibration response measurement.

The results presented in Figure 6 show the measured responses of the FUT from three different methods of dynamic characterisation. Since the measurement techniques utilise different sensor configurations, the vibration responses are recorded in terms of different physical parameters. The acoustic microphone measurements are collected where voltage and pressure are in direct proportion. For clarity, the pressure sign is provided to show the oscillating nature of the measured ultrasound signal. The laser Doppler vibrometer measures velocity through the Doppler effect, where again the velocity sign is shown to indicate the oscillatory motion, and the FUT_R_ measures sound energy via a conversion to electrical energy, where the receive voltage measured by the FUT_R_ requires amplification, since the sensitivity of the device is relatively low compared to that of either the acoustic microphone or the LDV system. In Figure 6, there is no significant over-shoot in the amplitude-time responses of the FUT measured at 40 kHz from each method, confirming that the FUT is being driven close to resonance. Clear over-shoot of the vibration amplitude is exhibited for each measurement technique for a drive frequency of 44 kHz, also displayed in Figure 6. However, the results shown in Figure 5 demonstrated that 40 kHz is a marginally off-resonance drive frequency, and so there will exist a small over-shoot in the amplitude-time spectrum. This is most prominently identified in the results from the acoustic microphone measurements, as exhibited in Figure 6a, where the response is not precisely at steady-state. The reason that this behavior is more conspicuous in this data set is due to the scaling of the ordinate axes.

**Figure 7:**
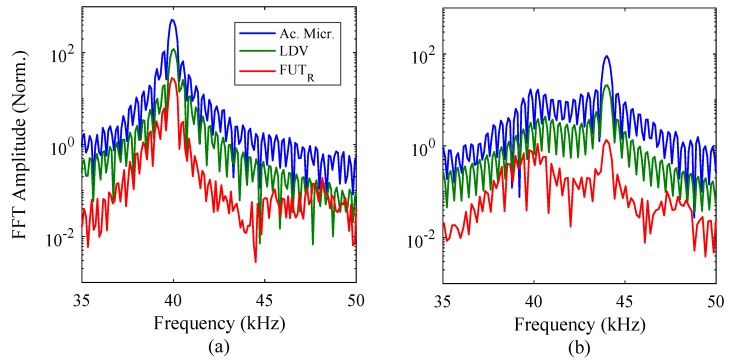
FFTs of the response signal for each measurement technique, for (**a**) 40 kHz and (**b**) 44 kHz burst sine drive signal excitations, each of 110 cycles.

Prior to the application of the mathematical model in the analysis of the results shown in Figure 6, the sensor results can be processed to provide a comparison of the measured resonance between each technique. FFTs were computed for all measurements from the three techniques, using a rectangular window and normalised to the mean square amplitude in each case. The FFT results are shown in Figure 7.

For a drive frequency of 40 kHz, as shown in Figure 7a, the measured frequency response from the three techniques correlate closely. The amplitudes of each data set have not been adjusted, to give an indication of the relative magnitudes of the FFTs in each case. The peaks in the amplitude-frequency spectrum for each measurement technique provide information regarding the drive signal frequency and the resonance frequency of the FUT. In the results shown in Figure 7a, it has been shown that the drive signal frequency and the resonance frequency of the FUT are close, and each measurement result is consistent with those of the other methods. The analysis of the 44 kHz drive signal shows a similar level of correlation. The energy in the amplitude-frequency spectrum is shared between the natural resonance around 40 kHz, and the drive signal frequency at 44 kHz.

**Figure 8:**
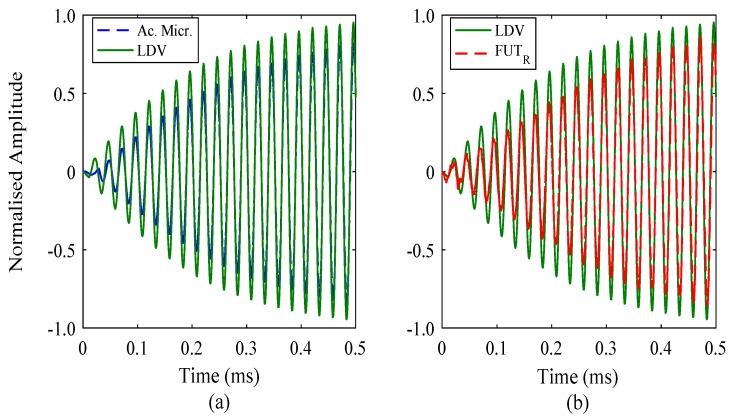
Initial region amplitude-time responses for the three measurement techniques, for a 40 kHz burst sine drive signal excitation of 110 cycles, showing the comparison between data from (**a**) the acoustic microphone and LDV, and (**b**) the LDV and FUT_R_. Note that the signals have been time shifted relative to each other to aid comparison of the profiles of the measured responses.

Despite this high level of correlation, there is not a precise overlap in the FFT computation for each case. Minor differences in the FFT spectra are important for the analysis of the initial region response of FUTs, since the amplitude rises from zero and is constantly changing until steady-state is reached. This means that the derived mathematical function should be able to predict the behaviour for a sinusoidal case. However, if the vibration motion is not instantaneously sinusoidal, then there may be a discrepancy with the mechanical analog model. This could have implications for how FUT-based systems are operated, particularly for a high number of FUT elements. Each measurement technique relies on a different sensor configuration. The LDV system employs an optical method utilising the Doppler effect, not a capacitive element such as that found in the acoustic microphone. Furthermore, the vibration response of the FUT_R_ is also affected by the flexural cap motion, the membrane of which is not assumed to instantaneously vibrate sinusoidally in response to a sinusoidal input signal. This is due to the mass inertia of the system, which does not affect a measurement process such as LDV. For these reasons, and to gain some insight into how the initial region response is generated and measured, the amplitude-time responses from each measurement technique were superimposed, with the results set to be in-phase towards the steady-state region. The purpose of this was to be able to identify any minor differences in vibration response, particularly at the time immediately after the drive signal is applied. The results are shown in Figure 8 for a 40 kHz and 110 cycle drive signal.

There is no substantial difference in terms of the measured signal shape between the responses collected by the acoustic microphone and LDV. A similar outcome has been found for the comparison between LDV and the FUT_R_, and through association, it has been demonstrated that the measured signal shape can generally be considered as consistent for the three measurement techniques. This is applicable to both the initial and steady-state region responses. The amplitudes of the measured signals have been normalised, but amplitude depends on the sensitivity of the measurement device, and so should not be used to correlate or compare measurement techniques. The close correlation of signals between that of the acoustic microphone and the FUT_R_ is not unexpected, due to the similarity in the way sound vibrations are recorded using these systems, with a flexing or bending membrane, as opposed to an optical method which the LDV system employs. However, the LDV measurements are near-field, whereas the acoustic microphone and FUT_R_ results are obtained in the far-field. More specifically, the acoustic microphone is a far-field wide-band device, and the FUT_R_ is a far-field narrow-band device, and therefore it is particularly interesting that the three measurement techniques appear to produce amplitude-time spectra of close correlation. The results show the suitability of all three techniques for high-quality vibration response measurement and characterisation, and that the measured response spectra are all similar, irrespective of the adopted measurement technique.

### 3.3. Mechanical Analog Model of the Initial Region

The mathematics of the steady-state and resonant ring-down regions of the transducer response are not reported here, being commonly accepted relationships [3,5]. However, a mathematical treatment of the initial response region has not been reported in detail. In general, a FUT can be considered as a lightly-damped resonator, with a mass M, damping factor C, and stiffness K, with a time-dependent forcing function which is assumed to be sinusoidal, that is shown by Fsin (*ωt*), with an angular drive frequency of ω. It is assumed that the FUT oscillates with a specific time-dependent vibration amplitude, from zero at rest, until a discrete time after which the steady-state response can be observed. Upon cessation of the drive signal, the FUT response decays at resonance. The steady-state and decay regions can be described by familiar mathematical relationships, and the initial region response prior to steady-state can be considered as an impulse signal which is used to drive the FUT to resonance. The switch from a rest condition to that of steady-state vibration is postulated to closely represent the effect of a unit step function. Since the response of the FUT switches from rest to the sinusoidal forcing function condition, the approach which has been used to develop the mathematical analog is through the convolution of the Heaviside step function and a sinusoidal forcing function of the form Fsin (ωt), where F is the amplitude of vibration. The basic equation for this convolved relationship is given by (1).

(1) $$M\ddot{x}+C\dot{x}+Kx=Fsin\;\left(\omega t\right)\cdot H\left(t_{0}-t\right)$$

Here, $t_{0}$ is the switch-off time of the forcing function, with H(t), the Heaviside function. The initial conditions are that both x and $\dot{x}$ are zero at t=0. The solution to (1) for $t<t_{0}$ is shown in (2), with the first and second derivatives shown in (3) and (4).

(2) $$x=F_{+}e^{\lambda_{+}t}+F_{-}e^{\lambda_{-}t}+Asin\;\left(\omega t\right)+Bcos\;\left(\omega t\right)$$

(3) $$\dot{x}=\lambda_{+}F_{+}e^{\lambda_{+}t}+\lambda_{-}F_{-}e^{\lambda_{-}t}+A\omega cos\;\left(\omega t\right)-B\omega sin\;\left(\omega t\right)$$

(4) $$\ddot{x}=\lambda_{+}^{2}F_{+}e^{\lambda_{+}t}+\lambda_{-}^{2}F_{-}e^{\lambda_{-}t}-A\omega^{2}sin\;\left(\omega t\right)-B\omega^{2}cos\;\left(\omega t\right)$$

The first and second terms in (2) are homogeneous which represent the natural resonance of the FUT, and the third and fourth terms together describe the forced excitation, or drive signal. The general solution of (1) can be formed, since H = 1 for 0 < *t* ≤ *t*_0_, and is shown in (5).

(5) $$M\ddot{x}+C\dot{x}+Kx=Fsin\;\left(\omega t\right)$$

Comparing (2) to (4) with (5), and collecting the exponential terms, gives (6). This equation can be solved to provide expressions for the $\lambda_{+}$ and $\lambda_{-}$ terms, shown in (7). Collecting the sin (ωt) and cos (ωt) terms gives expressions for A and B, shown in (8) to (13).

(6) $$M\lambda_{\pm }^{2}+C\lambda_{\pm }+K=0$$

(7) $$\lambda_{\pm }=\;\frac{-C\;\pm \;\sqrt{C^{2}-4MK}}{2M}$$

(8) $$B\left(-M\omega^{2}+K\right)+C\omega A=0$$

(9) $$A\left(-M\omega^{2}+K\right)-C\omega B=F$$

(10) $$B=\frac{C\omega A}{M\omega^{2}-K}$$

(11) $$A\left(-M\omega^{2}+K\right)-C\omega \left(\frac{C\omega A}{M\omega^{2}-K}\right)=F$$

(12) $$A\left(-M\omega^{2}+K\right)\left(M\omega^{2}-K\right)-C^{2}\omega^{2}A=F\left(M\omega^{2}-K\right)$$

(13) $$A=\frac{F\left(M\omega^{2}-K\right)}{\left(-M\omega^{2}+K\right)\left(M\omega^{2}-K\right)-C^{2}\omega^{2}}=gF$$

In order to calculate the amplitudes of the natural resonances, $F_{\pm }$, it is necessary to consider in a little more detail the terms $\lambda_{\pm }$. Applying the initial conditions that x and $\dot{x}$ are zero at t=0 to (2) and (3) gives (14) to (19).

(14) $$F_{+}+F_{-}+B=0$$

(15) $$F_{-}=-B-F_{+}$$

(16) $$\lambda_{+}F_{+}+\lambda_{-}F_{-}+A\omega =0$$

(17) $$\lambda_{+}F_{+}=-\lambda_{-}F_{-}-A\omega$$

(18) $$\lambda_{+}F_{+}=\lambda_{-}B+\lambda_{-}F_{+}-A\omega$$

(19) $$F_{+}\left(\lambda_{+}-\lambda_{-}\right)=\lambda_{-}B-A\omega$$

Using (7), the $\lambda_{+}$ and $\lambda_{-}$ terms can be expressed by (20) and (21). Subtracting these terms gives (22), enabling $F_{+}$ and $F_{-}$ to be determined, shown by (23) and (24).

(20) $$\lambda_{+}=\frac{-C+\;\sqrt{C^{2}-4MK}}{2M}$$

(21) $$\lambda_{-}=\frac{-C-\sqrt{C^{2}-4MK}}{2M}$$

(22) $$\left(\lambda_{+}-\lambda_{-}\right)=\;\frac{\sqrt{C^{2}-4MK}}{2M}$$

(23) $$F_{+}=\;\frac{B\left(\frac{-C-\;\sqrt{C^{2}-4MK}}{2M}\right)-A\omega }{\left(\frac{\sqrt{C^{2}-4MK}}{2M}\right)}$$

(24) $$F_{-}=\frac{\left(\frac{-C+\sqrt{C^{2}-4MK}}{2M}\right)F_{+}+A\omega }{-\left(\frac{C-\sqrt{C^{2}-4MK}}{2M}\right)}$$

The experimental results in Figure 6, and the prior investigations into the electro-mechanical behaviour of FUTs [14], indicate that the FUT resonates in a lightly damped, or underdamped, state. There are three distinct cases for the system damping, shown in (25) to (27).

For the overdamped case, $C^{2}>4MK$: $\lambda_{\pm }=\frac{-C\pm \bar{C}}{2M}$, where

(25) $$\lambda_{+}=\frac{-C+\bar{C}}{2M};\;\lambda_{-}=\;\frac{-C-\bar{C}}{2M}$$

For the critically damped case, $C^{2}=4MK$:

(26) $$\lambda_{+}=\lambda_{-}=-\frac{C}{2M}=\;-\alpha$$

For the underdamped case, $C^{2}<4MK$: $\lambda_{\pm }=\frac{-C\;\pm \;i\bar{C}}{2M}$, where $\lambda_{+}=\;\frac{-C+i\bar{C}}{2M}$; $\lambda_{-}=\;\frac{-C-i\bar{C}}{2M}$;

(27) $$\alpha =\frac{C}{2M};\;\bar{\alpha }=\frac{\bar{C}}{2M};\;\bar{C}=\sqrt{4MK-C^{2}}$$

The FUT is assumed to be operating in an underdamped state, being a resonant transducer. The underdamped case of oscillation, analogous to that produced in the response of the FUT, results in a complex term in the response equation. The solution to the equation of motion for the FUT can then be written as shown in (28) to (30), using standard trigonometric identities.

(28) $$x\left(t\right)=F_{+}e^{\left(-\alpha +i\bar{\alpha }\right)t}+F_{-}e^{\left(-\alpha -i\bar{\alpha }\right)t}+Asin\left(\omega t\right)+Bcos\left(\omega t\right)$$

(29) $$x\left(t\right)=e^{-\alpha t}e^{i\alpha t}F_{+}+e^{-\alpha t}e^{-i\alpha t}F_{-}+Asin\left(\omega t\right)+Bcos\left(\omega t\right)$$

(30) $$x\left(t\right)=F_{+}e^{-\alpha t}\left\{\left(cos\bar{\alpha }t\right)+\left(isin\bar{\alpha }t\right)\right\}+F_{-}e^{-\alpha t}\left\{\left(cos\bar{\alpha }t\right)-\left(isin\bar{\alpha }t\right)\right\}+Asin\left(\omega t\right)+Bcos\left(\omega t\right)$$

With respect to the drive signal, the A and B parameters are related to the forced excitation amplitude once the FUT drive signal is switched on. The amplitude response to the drive signal in this initial region of the FUT response is denoted by $\bar{E}$. The solution to (1), and the above results, can be used to derive the amplitude $\bar{E}$, which is represented by $\sqrt{A^{2}+B^{2}}$, and phase Φ parameters for the FUT response under forced excitation approaching steady-state, shown in (31), which can be used in the computation of the response as a function of time. Using (10) and (13), an expression for $\bar{E}$ can be derived, which is shown in (32) to (35).

(31) $$x\left(t\right)=F_{+}e^{-\alpha t}(\cos\bar{\alpha }t+isin\bar{\alpha }t)+F_{-}e^{-\alpha t}(\cos\bar{\alpha }t-isin\bar{\alpha }t)\;\;+\sqrt{A^{2}+B^{2}}\left(\sin\left(\omega t+\Phi \right)\right)\;\text{where}\;tan\Phi =\frac{B}{A}$$

(32) $$A^{2}+B^{2}=A^{2}+A^{2}\left(\frac{C\omega }{M\omega^{2}-K}\right){\;}^{2}$$

(33) $$A^{2}+B^{2}={\left(gF\right)}^{2}\left\{1+\left(\frac{C\omega }{M\omega^{2}-K}\right){\;}^{2}\right\}$$

(34) $$A^{2}+B^{2}={\left(gF\right)}^{2}\left(\frac{{\left(M\omega^{2}-K\right)}^{2}+{\left(C\omega \right)}^{2}}{{\left(M\omega^{2}-K\right)}^{2}}\right)$$

(35) $$\bar{E}=\sqrt{A^{2}+B^{2}}=\frac{gF}{\left(M\omega^{2}-K\right)}\sqrt{{\left(M\omega^{2}-K\right)}^{2}+{\left(C\omega \right)}^{2}}$$

**Figure 9:**
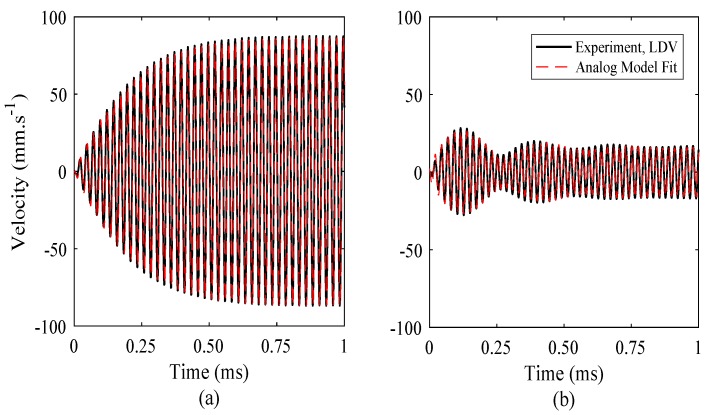
FUT vibration response in the initial region determined from the analog model compared with experimentally obtained LDV data, for drive frequencies of (**a**) 40 kHz and (**b**) 44 kHz. The experimental data and the fit of the mechanical analog model produced from (31) almost completely overlap in each case.

The derived parameters can be used to generate an amplitude-time spectrum which represents the build-up towards steady-state, for a specific drive frequency. Equation (31) can be adapted to generate the real part of the response only, which represents the vibration response of the FUT, as shown by (36). The responses for drive signals of 40 kHz and 44 kHz are shown in Figure 9, which are compared to the FUT response measured using LDV, from Figure 6.

(36) $$x\left(t\right)=\left(F_{+}+F_{-}\right)\left(e^{-\alpha t}\cos\bar{\alpha }t\right)+\bar{E}\left(\sin\left(\omega t+\Phi \right)\right)$$

The results show a close correlation between the numerical and experimental data, demonstrating the reliability of the mechanical analog model to accurately predict the vibration response of the FUT, and for different drive frequencies. It also provides insight into the mechanisms of the dynamic response of a FUT, which is invaluable in the future design and optimisation of transducers based on this configuration. In general, it is anticipated that this model will be used in future to simulate the performance of a FUT, and will aid in the design process in order to tailor FUT designs for particular industrial or medical applications that utilise different drive frequencies to improve their performance.

## 4. Conclusions

Although low frequency FUT devices have been commercially available for decades, there is remarkably little in the published literature to explain their operation or demonstrate how their performance can be thoroughly analysed using direct measurements of the flexural membrane and the resulting ultrasonic disturbances generated in a fluid. This study has demonstrated the complex dynamic performance of FUTs for the first time, validated through three different methods of vibration response measurement. Detailed experimental investigation has shown the viability of acoustic microphone, laser Doppler vibrometry, and FUT sensor measurements in the identification of the multi-region vibration response of a FUT. These results show that the physics of the vibration response can be examined irrespective of characterisation method, where each incorporates the necessary measurement sensitivity. Furthermore, the experimental investigations have enabled the development of a robust mechanical analog model that has not been described or published previously for a FUT, with the derivation presented in detail. It has been established that analytical results closely correlate with experimental data. The analog model itself is not a physical model of the transducer behaviour, but demonstrates that the transducer response is very well described by that of the simplified mechanical analog model suggested in this study. The outcomes of this research will be invaluable in the wider industrial application of FUTs, and will enable the prediction of how real transducers with unavoidable, slightly different mechanical responses will behave in experimental conditions and applications. This will in turn facilitate the design of methods that can be used to work with the realistic variations and limitations of these highly efficient ultrasonic transducers.
